# Case Series of Mandibular Glandular Odontogenic Cysts: Radiographic and Histopathological Evaluation

**DOI:** 10.1155/crid/1637523

**Published:** 2025-08-18

**Authors:** Hanadi Sabban, Nagla'a Abdel-Wahed, Hanan F. AbdelMaguid, Suwarna Dangore- Khasbage, Yasmin Mair, Hanadi Khalifa, Raghd Alansari, Hisham Abbas Komo

**Affiliations:** ^1^Oral Diagnostic Sciences Department, King Abdulaziz University Faculty of Dentistry, Jeddah, Makkah Province, Saudi Arabia; ^2^Oral Radiology Department, Faculty of Dentistry, Cairo University, Cairo, Egypt; ^3^Department of Oral Medicine & Radiology, Sharad Pawar Dental College & Hospital, Datta Meghe Institute of Higher Education and Research (Deemed to Be University), Wardha, Maharashtra, India; ^4^Radiology Department, King Abdulaziz University Dental Hospital, Jeddah, Makkah Province, Saudi Arabia; ^5^Oral and Maxillofacial Surgery Department, King Abdulaziz University Dental Hospital, Jeddah, Makkah Province, Saudi Arabia

**Keywords:** CBCT, cone beam CT, cone beam computed tomography, glandular odontogenic cyst, histopathology, jaw, mandible, mandibular cyst, radiographic features, radiographic image interpretation

## Abstract

Glandular odontogenic cysts (GOCs) have been previously documented in the literature as uncommon odontogenic cysts characterized by their aggressive nature and high recurrence rate. This study is aimed at documenting and analyzing the radiographic as well as the histopathological features of GOC in the mandible and correlating these characteristics to previously reported studies. This case series includes five male patients, aged between 32 and 50 years, who were interpreted using cone beam CT (CBCT) scans at the Oral Radiology Department of King Abdulaziz University Dental Hospital between 2022 and 2023. All cases were reported histopathologically as GOC. The reported lesions were all located in the mandible, with three in the anterior region and two in the posterior region. Two lesions extended across the midline. Radiographically, two lesions were unilocular and three were multilocular, with straight, long septa intersecting at right angles to the outer border. The lesions exhibited well-defined, corticated borders with scalloping between roots. Effects on adjacent structures included thinning of the buccal/lingual cortical plates, expansion, inferior displacement of the mandibular canal, and tooth displacement, and one case showed loss of lamina dura, root resorption, and cortical border perforation. The microscopic examination of each case is described. Finally, GOC presents radiographic features similar to other odontogenic and neoplastic lesions such as odontogenic keratocyst, ameloblastoma, lateral periodontal cyst, residual cyst, and central giant cell granuloma. Accurate diagnosis requires careful histopathological examination and long-term follow-up to confirm the diagnosis and monitor for potential recurrences.

## 1. Introduction

Glandular odontogenic cyst (GOC) of the mandible has been previously documented as an uncommon cyst of odontogenic origin, primarily affecting the mandible. It was described in 1988 by Gardner et al., GOC as characterized by its diverse histopathological features, including glandular structures, mucous cells, and squamous epithelium. Its diagnosis poses challenges due to its histological similarities with other cystic lesions, such as mucoepidermoid carcinoma and lateral periodontal cysts [1]. According to the literature, diagnosing and treating GOC can be challenging, especially when it occurs in the mandible. One of the most comprehensive studies of GOC was presented by Fowler et al., who studied 46 cases and focused on the microscopic specifications necessary for diagnosis. Mucous cells, ciliated cells, and a lining epithelium of varying thickness are among the histological variabilities that were highlighted in their investigation [2]. Given these varied characteristics, it is important to make a careful differential diagnosis in order to differentiate GOC from other cystic entities, especially mucoepidermoid carcinoma, which may present similarly. The authors stressed the value of immunohistochemical staining in supporting a precise diagnosis of GOC, pointing out that markers such as p63, CK7, and CK19 can be helpful in distinguishing it from other lesions [3]. The study draws attention to the difficulties in handling these situations, where a benign diagnosis may turn into a complicated clinical situation that calls for interdisciplinary care. New case reports continue to clarify the histopathological and clinical range of GOC, especially in the mandible. A case of GOC in the mandible was presented by Udompatanakorn et al., who focused on the radiographic characteristics of the cyst and its surgical treatment [4]. Their report emphasized how GOC appears on imaging as radiolucent and well defined, frequently resembling other odontogenic cysts. Although the authors pointed out that a comprehensive histopathological examination is crucial to ensuring an accurate diagnosis and ruling out more aggressive pathologies, surgical enucleation is still the preferred treatment. A rare instance of a posterior mandibular glandular cyst was also reported by Kochaji et al., adding to the small pool of GOC instances in this anatomical area that have been documented [5]. Their case highlighted the diagnostic difficulties associated with GOC, especially when it comes to differentiating it from other jaw cystic lesions. The condition is uncommon in the posterior mandible, which makes diagnosis more difficult and calls for a thorough histopathological analysis and a high index of suspicion. In conclusion, the literature highlighted the difficulties in diagnosing GOCs, especially those in the mandible, as well as the high rate of recurrence. In order to underscore the significance of a precise diagnosis of this aggressive lesion in the future, this case series is aimed at providing important insights into the radiographic and histopathological features of the GOC.

## 2. Case Presentation

Five cases are presented in this case series study, who were referred for radiographic interpretation by cone beam computed tomography (CBCT) imaging modality at the Oral Radiology Department in the King Abdulaziz University Dental Hospital between 2022 and 2023. All the five patients had been diagnosed with GOCs as explained and highlighted in this literature.

Imaging protocol: Imaging was performed using an i-CAT Imaging Sciences International Inc. (Hatfield, Pennsylvania, United States) device for four patients and a Cranex Novus e device (Soredex, Helsinki, Finland) for one patient. The i-CAT device had a field of view (FOV) of 17 × 23 cm, while the Cranex Novus e operated at 90 kV and 10 mA with a CCD sensor. The FOV was adjusted to include the entire area of interest, with a voxel size of 0.25 mm for i-CAT and 0.3 mm for Cranex Novus e. OnDemand3D software (CyberMed, Seoul, South Korea) was used for radiographic interpretation of the scans. The scans were reviewed by two experienced oral radiologists to assess the radiographic features of the lesions and reported as mentioned in the following paragraphs. The radiographic features of each GOC case were documented to describe the lesion size, location, margin outlines, internal structures, effect on the surroundings such as root resorption or cortical bone involvement, and significant radiographic features such as bone pattern and crossing the midline. Biopsies of these lesions were taken and examined histopathologically by board-certified pathologists.

### 2.1. Case 1

**Figure 1:**
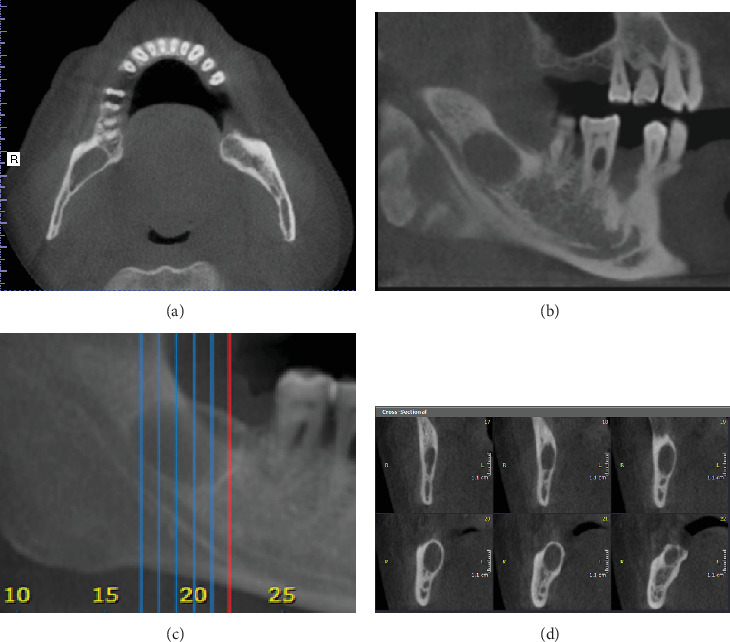
(a) Axial, (b) corrected sagittal, (c) reconstructed panorama, and (d) cross-sectional images of constructed panorama are showing a well-defined, well-corticated unilocular radiolucent lesion. There is thinning of the lingual cortical plate adjacent to the lesion and subtle expansion lingually; yet, there is evident expansion superiorly and fusiform anteroposterior expansion.

A 50-year-old male presented with a complaint of multiple loose teeth. Radiographic examination incidentally revealed a lesion in the right posterior mandible, located in the area of the missing Tooth #48 and retromolar region. The lesion appeared as a well-defined, corticated, unilocular radiolucency, with dimensions of 1.5 cm in height, 1 cm in buccolingual length, and 2 cm in anteroposterior length. There was thinning of the lingual cortical plate adjacent to the lesion, with subtle lingual expansion and notable superior expansion. The lesion may have caused inferior displacement of the right mandibular canal, with a fusiform expansion in the anteroposterior dimension. The differential diagnosis included residual cyst, odontogenic keratocyst (OKC), and unicystic ameloblastoma (Figures 1a, 1b, 1c, and 1d). The fact that the patient could not recall whether or not the third molar had been extracted or not debased the residual cyst among the differential diagnosis list. This, along with the minimal buccolingual expansion and the fusiform anteroposterior expansion, made the first suggested possibility in favor of OKC.

**Figure 2:**
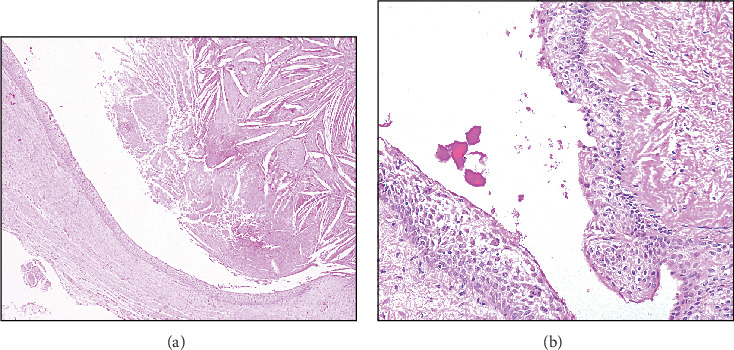
(a) GOC showing cholesterol clefts granuloma in the lumen (4X). (b) The epithelial lining shows whorling, snouting (20X).

A biopsy was taken. The microscopic examination reveals multiple fragments of a cystic lesion lined by nonkeratinized epithelium with variable thickness. The lining varies from cuboidal cells to stratified squamous epithelium with some areas of whorling and snouting. Focal areas of papillary projections, cilia, and mucous cells are noted. The cystic wall consists of fibrous connective tissue with interspersed fibroblasts and delicate blood capillaries. In addition, cholesterol clefts surrounded by giant cells are present along the border of the cystic lumen (Figure 2a,b).

### 2.2. Case 2

**Figure 3:**
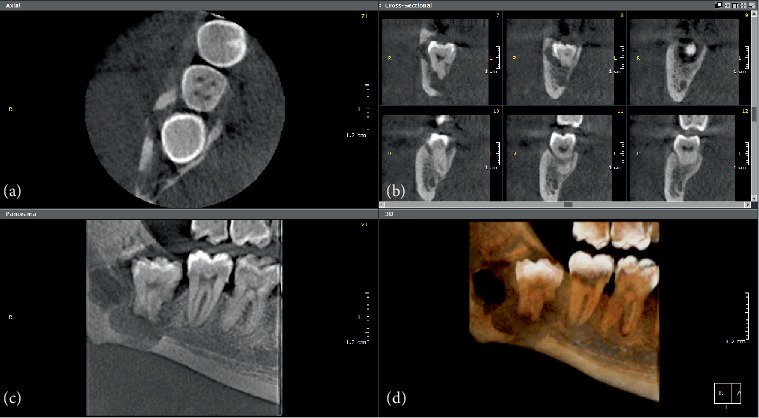
(a) Axial, (b) cross-sectional, (c) reconstructed panorama, and (d) 3D rendering images are showing a well-defined, partially corticated, multilocular radiolucency associated with impacted #48. It occupies the whole buccolingual width. There is loss of the lamina dura and root resorption of #48. There is disruption in the lingual cortex, inferior border of the mandible, and the cortices of the respective IANC.

A 32-year-old male presented in the surgery clinic with dull, aching pain that often exacerbates into severe shooting pain into the lower right molar area. Radiographic examination revealed an entity extending from distal to Tooth #47 to the ascending ramus and from the alveolar crest to the inferior border of the mandible, occupying the entire buccolingual width. The lesion presented radiographically as a well-defined, partially corticated multilocular radiolucency associated with impacted Tooth #48, featuring thin septa and large locules. Notable findings included loss of the lamina dura and root resorption of Tooth #48, as well as disruption (perforation) in the lingual cortex, inferior border of the mandible, and the cortices of the inferior alveolar nerve canal (IANC). Additionally, there was a discontinuity in the facial/buccal aspect of the ramus buccal to Tooth #48, consistent with a sinus tract. The bone pattern in the mandible appeared sclerotic, likely due to chronic inflammation. The radiographic interpretation included aggressive benign odontogenic tumors such as ameloblastoma or low-grade mucoepidermoid carcinoma (Figures 3a, 3b, 3c, and 3d).

**Figure 4:**
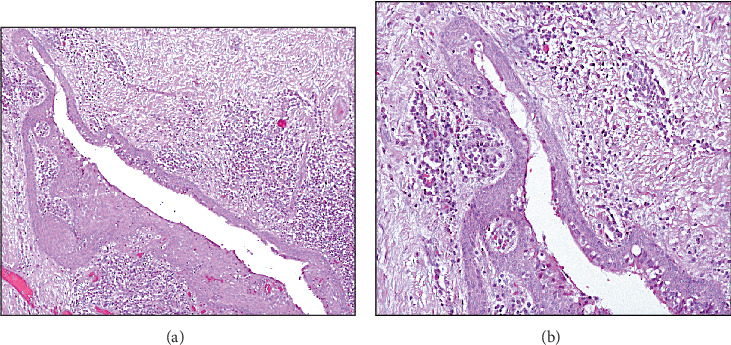
(a) GOC with inflamed cystic wall (4X). (b) The lining consists of nonkeratinized squamous epithelium with variable thickness and microcystic spaces (20X).

The microscopic examination revealed multiple fragments of a cystic lesion lined by epithelium of nonkeratinized flattened squamous to cuboidal cells, with some areas of whorling. Other sections showed spongiotic epithelial lining and arcading of the rete ridges. Other areas showed glandular features including papillary projection, cilia, microcystic spaces, and mucous cells. The cystic wall consisted of fibrous connective tissue with extensive inflammation and focal SOT-like features. Bacterial colonies and areas of hemorrhage were seen in the examined section, which was consistent with the patient's chief complaint of the pain, which translated into a secondary infection of a chronic lesion. A diagnosis of an inflamed GOC was made. This was interpreted radiographically as loss of cortication as well as granular/sclerotic appearance at the adjacent bone structure in the mandible (Figure 4a,b).

### 2.3. Case 3

**Figure 5:**
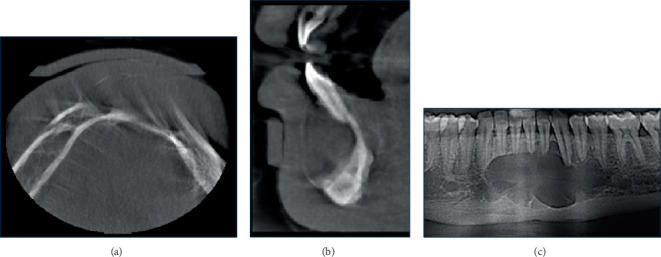
(a) Axial, (b) sagittal, and (c) reconstructed panoramic images of CBCT showing a well-defined, multilocular, radiolucent lesion. The root of Tooth #33 is displaced distally, and there is root resorption in Teeth #42 and #41. There is thinning, displacement, and possible perforation in the buccal cortical plate and thinning of the lingual cortical plate.

A 32-year-old male presented with a swelling in the lower anterior area. Radiographic examination revealed a lesion extending from Tooth #35 to Tooth #43 and from the root apices to the inferior third of the mandible. This lesion is well defined, multilocular, and radiolucent with scalloping borders. The CBCT scan showed displacement of the root of Tooth #33 distally, along with thinning, displacement, and potential perforation of the buccal cortical plate, and thinning of the lingual cortical plate. Additionally, there was evidence of root resorption in Teeth #42 and #41. This lesion crossed the midline. The differential diagnosis included odontogenic cysts, possibly an OKC, and GOC. Upon clinical examination, the vitality of Teeth #33 to #43 was noted (Figures 5a, 5b, and 5c).

**Figure 6:**
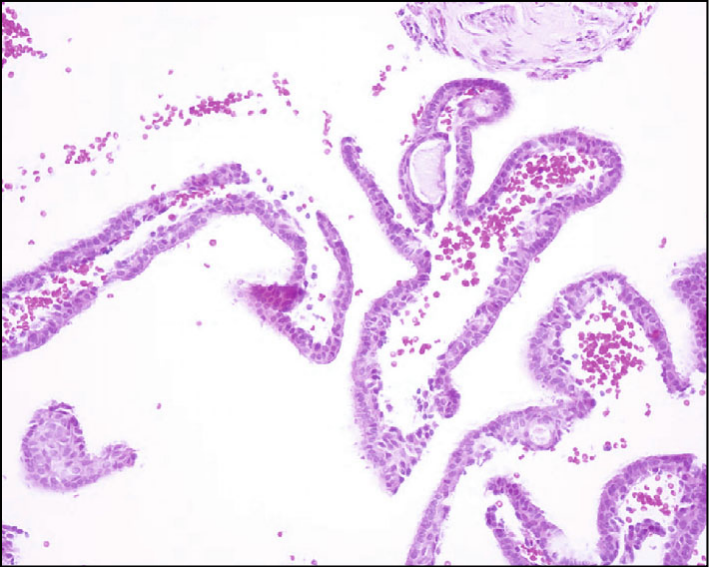
Detached epithelial lining consists of two layers of cuboidal and columnar cells with microcystic spaces. Note the connective tissue wall.

Histopathological examination confirmed the diagnosis of a GOC. The histopathology reveals a specimen with multiple fragments of soft tissue showing a cystic lesion. The cyst lining is formed by cuboidal to columnar cells and is detached in some areas, showing microcystic spaces. In addition, stratified squamous epithelial lining is present exhibiting focal spherical plaque and mucous goblet cells. Furthermore, luminal cells in localized areas demonstrate hobnail configuration with the presence of cilia. The fibrous connective tissue wall contains blood capillaries and dispersed chronic inflammatory cells (Figure 6).

### 2.4. Case 4

**Figure 7:**
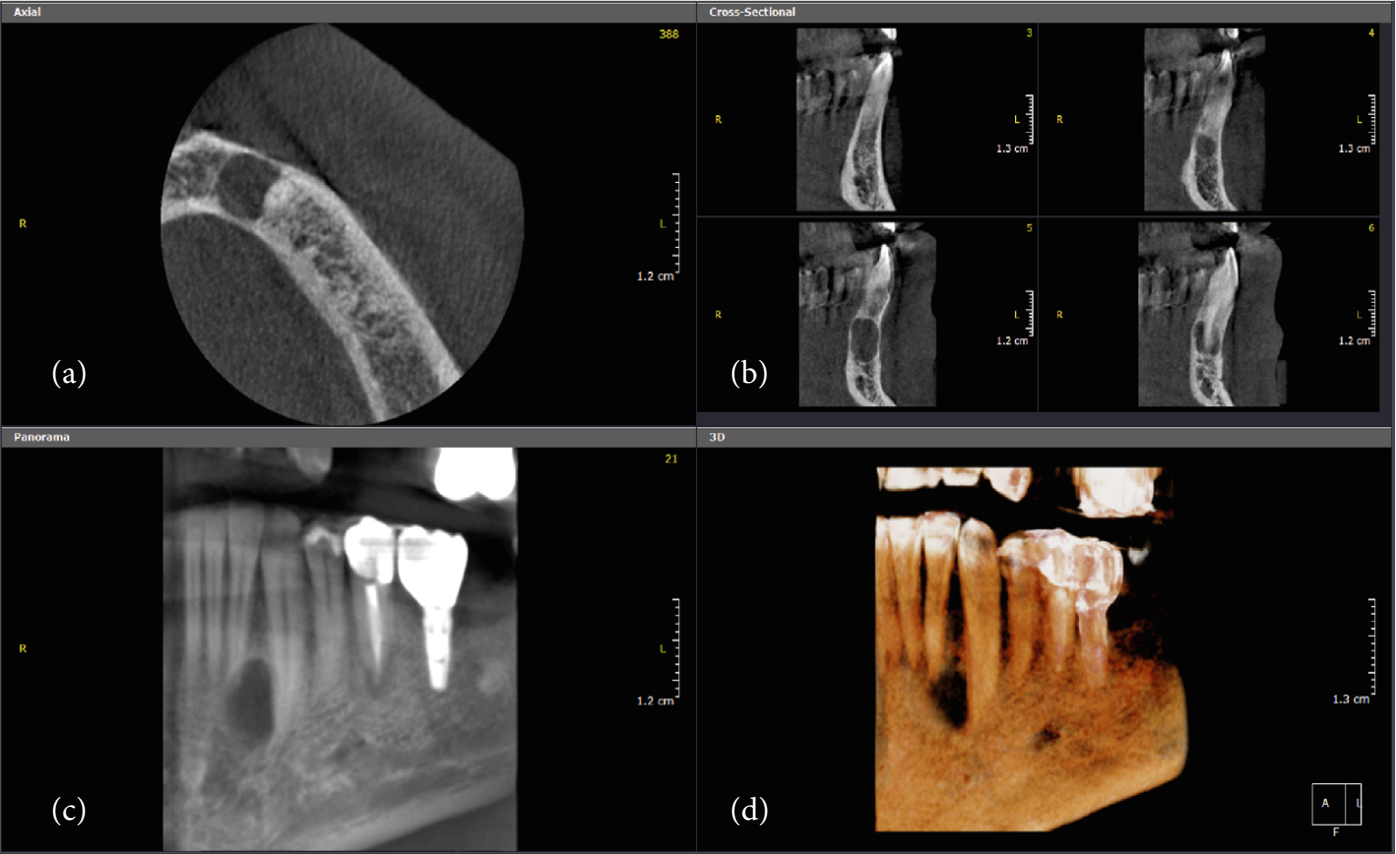
(a) Axial, (b) cross-sectional, (c) reconstructed panorama, and (d) 3D rendering images are showing a well-defined, radiolucent lesion between the roots of Teeth #32 and #33. There is loss of the lamina dura in the associated teeth. There is thinning of the buccal and lingual cortical plates with slight buccolingual expansion.

A 34-year-old male patient visited the clinic with a “little discomfort in the lower left anterior region.” Radiographic examination revealed a lesion extending between the roots of Teeth #32 and #33, measuring around 1.1 cm in height, 0.8 cm in buccolingual dimension, and 0.8 cm in anteroposterior dimension. The lesion was well defined, corticated, and appeared as a unilocular radiolucency. There was thinning of both the lingual and buccal cortical plates, with mild expansion observed lingually. The differential diagnosis included lateral periodontal cyst and OKC (Figures 7a, 7b, 7c, and 7d). Clinically, the adjacent teeth tested vital.

**Figure 8:**
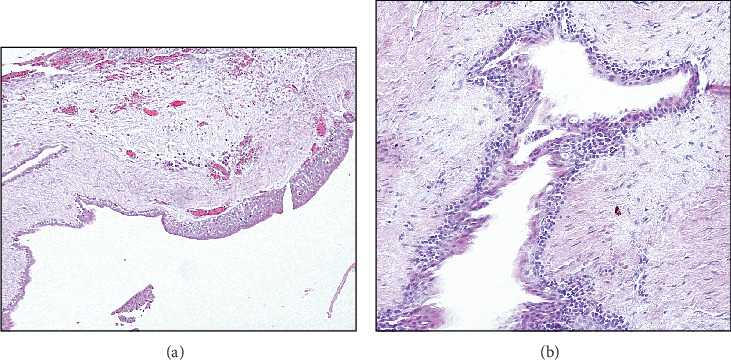
(a) Variable thickness of the epithelial lining (4X). (b) Higher magnification shows ductlike structure of the epithelium (20X).

The histopathological examination of the section consisted of a cystic cavity lined by a nonkeratinized stratified squamous epithelium of variable thickness supported by a fibrous connective tissue wall. The epithelial lining shows duct-like spaces, mucous cells, and luminal eosinophilic cells with hobnailing and snouting. The connective tissue wall shows collagen fibers, mild inflammatory infiltrate mainly lymphocytes, and extravasated red blood cells (Figure 8a,b).

### 2.5. Case 5

**Figure 9:**
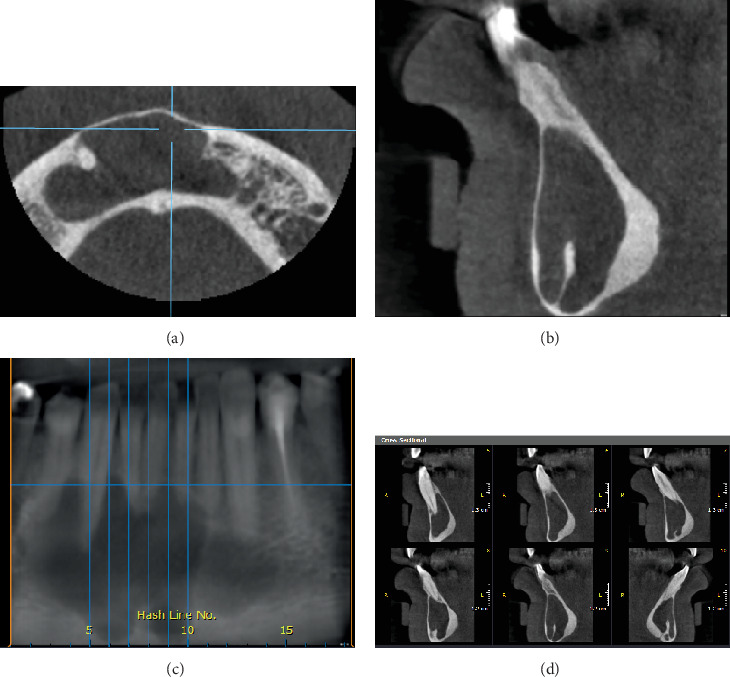
(a) Axial, (b) sagittal, (c) reconstructed panorama, and (d) cross-sectional images are showing a well-defined, corticated, multilocular radiolucent lesion in the anterior mandible. It scallops between the roots and has caused slight labial expansion as well as displacement of the roots of #41 and #42. At the inferior aspect of the lesion, there is coarse curved septation giving the lesion the multilocular appearance.

**Figure 10:**
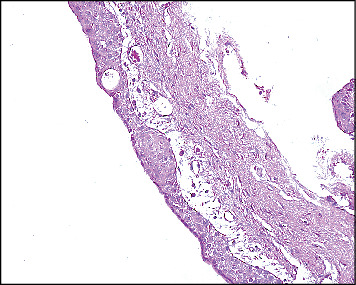
The cyst is lined by nonkeratinized stratified squamous epithelium with whorling and a ductlike space (20X).

A 47-year-old male presented with a swelling in the anterior mandible. Radiographic examination revealed a lesion extending from the periapical area of Tooth #44 to the periapical area of Tooth #32 and from the midroot level to the inferior border of the mandible. It was well defined with corticated, scalloping borders and appeared as a multilocular radiolucency with low density. Notably, the inferior aspect of the lesion displayed coarse, curved septation. The lesion scalloped between the roots, causing slight labial expansion and displacement of the roots of Teeth #41 and #42. It extended across the midline and featured coarse curved septa intersecting at right angles to the outer border (Figure 9). The differential diagnosis included OKC, GOC, simple bone cyst, and central giant cell granuloma (CGCG). An incisional biopsy was performed, whereby the microscopic examination revealed a cystic lesion lined by nonkeratinized stratified squamous epithelium with whorling. Focal areas of duct-like structure were noted. The underlying connective tissue wall consisted of collagen fibers with interspersed fibroblasts and delicate blood capillaries (Figure 10) Radiographic correlation of the case warranted the diagnosis of GOC.

### 2.6. Follow-Up and Outcome Assessment

**Figure 11:**
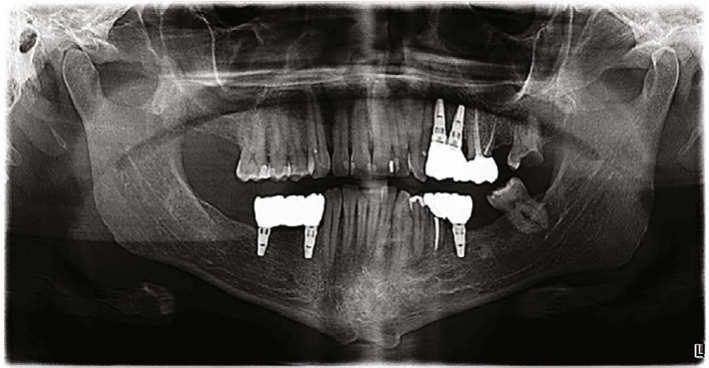
Panoramic radiograph of Case 4 taken 2 years postoperatively. In the left anterior mandible, between the roots of Teeth #32 and #33, there is a well-healed bone with no signs of recurrence. A thin radiolucent rim persists, likely representing residual healing.

All five cases in this series were diagnosed and managed at King Abdulaziz University Dental Hospital between 2022 and 2023. The treatment approach varied based on lesion characteristics and extent at the time of diagnosis:

• Cases 1–3 underwent cyst enucleation with peripheral curettage following confirmatory incisional biopsy. Despite scheduling routine follow-up visits, these patients did not return for clinical or radiographic evaluation, and no posttreatment imaging was available. It is noteworthy that these cases presented relatively recently, and given the typically slow-growing nature of odontogenic cysts and tumors, it is plausible that patients did not experience symptoms significant enough to prompt return visits, which may explain the absence of documented recurrences to date.
• Case 4 was managed via excisional biopsy. A postoperative CBCT scan—acquired for an unrelated diagnostic purpose—incidentally included part of the surgical site. The scan revealed incomplete healing with residual defects in the buccal and lingual cortical plates. However, a follow-up panoramic radiograph taken approximately 2 years later (Figure 11) demonstrated well-healed bone architecture and no radiographic evidence of recurrence, apart from the presence of a thin, radiolucent capsule consistent with residual postoperative remodeling.
• Case 5 received only an incisional biopsy at our institution and did not return for definitive treatment or follow-up. No subsequent records or radiographic data were available.

In summary, detailed follow-up data were only available for Case 4. The lack of recurrence in this case is reassuring; however, the absence of follow-up in the remaining cases represents a limitation. The relatively short duration since initial management and the asymptomatic nature of early recurrence may account for the patients' lack of return but emphasize the importance of structured recall protocols and long-term surveillance for lesions with known recurrence potential.

## 3. Discussion

This case series provides valuable insights into the radiographic features of GOCs based on a series of five cases. The findings offer a detailed comparison with previous literature and underscore the importance of accurate diagnosis and management. With a correlation between radiographic and histopathological features, the lesions were diagnosed as GOCs in the mandible. The radiographic features for the five cases showed that all cases were males and aged between 32 and 50 years. Among these cases, three lesions were located in the anterior mandible, and two were situated posteriorly. Two of the lesions extended across the midline, indicating a more extensive involvement. The radiographic examination revealed that two of the lesions were unilocular, while three were multilocular. The septa within the multilocular lesions were straight, long, and intersected at right angles to the outer border. The borders of the lesions were well defined and corticated, with scalloping observed between the roots. Surrounding structures were notably affected by these cysts, including thinning of the buccal and lingual cortical plates, expansion, inferior displacement of the mandibular canal, and displacement of adjacent teeth. One lesion also demonstrated loss of the lamina dura and root resorption, while another showed perforation of the cortical borders. These findings are consistent with the established radiographic features of GOC reported in the literature. The presence of well-defined, corticated borders, scalloping between the roots, and the potential for significant impact on adjacent anatomical structures reinforces the need for careful radiographic assessment in the diagnosis and management of GOC. The ability of these cysts to cross the midline and affect the mandibular canal further highlights their aggressive nature, underscoring the importance of early detection and intervention. Regarding Case 2, the radiographic features related to partial corticated border as well as sclerotic bone reaction surrounding the borders demonstrate the presence of a secondary infection which is confirmed histopathologically, since our case showed inflammation in the cystic wall.

Our study's observation of well-defined, corticated borders and scalloping between roots aligns with reports from Udompatanakorn et al. [4] and Kochaji et al. [5]. Udompatanakorn et al. emphasized the characteristic well-defined radiolucency of GOC, which is consistent with our findings of clear borders and scalloping. Kochaji et al. also highlighted similar radiographic features in a rare posterior mandibular case, reinforcing the need for a high index of suspicion in such lesions. The multilocular nature of some GOCs observed in this study supports previous findings by Maruyama et al. [6], who reported that GOC can present as multilocular lesions with a complex internal structure. This multilocular pattern, characterized by straight and long septa intersecting at right angles, has been documented by Tambawala et al. [7], indicating a common feature among GOC cases. Significant effects on adjacent structures such as thinning of the cortical plates, displacement of the mandibular canal, and tooth displacement were observed in our cases. These findings corroborate the observations of Manor et al. [8] and Kaplan et al. [9], who noted similar changes in their studies of GOC. The potential for such significant impact highlights the aggressive nature of GOC and its capacity to alter surrounding anatomy. This underscores the importance of long-term follow-up, as emphasized by Peraza-Labrador et al. [10], who stressed the need for extended monitoring to manage the risk of recurrence effectively. The challenges in differentiating GOC from other odontogenic and neoplastic lesions have been highlighted in the literature, including Gorgis et al. [11] and de Arruda et al. [12], who demonstrated that GOC can radiographically resemble central mucoepidermoid carcinoma or periapical inflammatory lesions, thereby necessitating careful histopathological evaluation for accurate diagnosis. Furthermore, the difficulty in distinguishing GOC from other odontogenic cysts or neoplasms—and its potential to perforate cortical bone—is well documented, as demonstrated by Espinoza-Ganchozo et al. [13], whose case involved multiple diagnostic mimics and clear evidence of cortical perforation. This rare feature further underscores the aggressive nature of the cyst and the need for vigilant diagnostic and management approaches. In conclusion, the radiographic and clinical features of GOC observed in this study are consistent with those reported in the literature. The overlap with other odontogenic lesions necessitates thorough histopathological examination and long-term follow-up to ensure accurate diagnosis and effective management. Integrating radiographic findings with histopathological confirmation remains crucial in addressing the challenges posed by GOC. In conclusion, GOCs exhibit radiographic features that can closely resemble those of other odontogenic lesions, such as OKCs, ameloblastomas, lateral periodontal cysts, residual cysts, mucoepidermoid carcinoma, and CGCGs. Due to this overlap in radiographic appearance, a careful histopathological examination is essential for accurate diagnosis for this aggressive lesion which could be misdiagnosed with secondary infection. Additionally, given the potential for recurrence, long-term follow-up is crucial in managing patients with GOC.

The current study highlights that diagnosing GOC is not possible from clinical and radiographic findings alone, since certain GOC microscopic features may be detected in radicular, dentigerous, botryoid cysts, and central mucoepidermoid carcinoma (CMEC). Histopathologic examination usually adds clarity in diagnosing the entity, but caution must be exercised due to the overlap of histopathologic findings with other lesions and lack of pathognomonic features [14].

In the current study, specimens from the five cases shared a histopathological picture of a cyst lined by nonkeratinized squamous epithelium with variable thickness and areas of whorling. In two of the cases, microcysts were evident upon microscopic examination. Papillary projections in the epithelial lining were seen in two cases. Mucoid/goblet cells were apparent in two cases, while cilia were present in three of the five cases. Multiple cysts were also evident in three cases. Hobnail cuboidal cells were seen in two of the examined specimens. Two cases revealed the presence of cholesterol clefts. Apocrine snouting was also reported in two of the examined specimens. Only one case showed bacterial colonies and areas of hemorrhage. Although the reported five cases had no pathognomonic features, they all showed some of the criteria of GOC described in previous literature.  Table 1 highlights the major and minor criteria listed by Kaplan et al. [9].  Table 2 shows the microscopic findings reported for the five cases in the current study.

Although immunohistochemistry (IHC) can be helpful in the differential diagnosis of cystic lesions of the jaws, especially when distinguishing GOC from low-grade mucoepidermoid carcinoma, it was not performed in this series. According to the fourth edition of the *World Health Organization (WHO) Classification of Head and Neck Tumors*, the diagnosis of GOC can be reliably established depending on distinct histopathological characteristics alone—such as nonkeratinized stratified squamous epithelium with variable thickness, intraepithelial microcysts or duct-like spaces, hobnail cells, and epithelial plaques. Regarding our cases, although two specimens showed focal goblet or mucous cells, none exhibited histologic features suspicious for mucoepidermoid carcinoma that would necessitate the use of IHC for confirmation [1, 2].

All cases were diagnosed and treated at King Abdulaziz University Dental Hospital between 2022 and 2023, but only one patient returned for documented follow-up. Although these lesions are well known with high recurrence rates reported in the literature, such as OKCs and ameloblastomas, no recurrence was observed in our study—likely due to the short follow-up period and lack of symptoms prompting patient return. This suggests the critical demand for long-term clinical and radiographic surveillance, particularly for lesions with aggressive behavior and high recurrence potential.

The presence of nonkeratinized stratified squamous epithelium of varying thickness, along with the areas of whorling reported in the cases of the current study, aligns with the findings reported by Gorgis et al., de Arruda et al., and Krishnamurthy et al. [11, 12, 15]. Epithelial whorls have also been reported in specimens of lateral periodontal cysts and botryoid cysts, indicating the odontogenic nature of the GOC. These areas of epithelial thickening resemble changes seen in the dental lamina [15].

Evidence of the presence of cholesterol clefts and epithelial spheres has also been reported in cases studied by Krishnamurthy et al. [15]. The presence of microcysts noticed in our study was also evident in cases studied by Gorgis et al., de Arruda et al., and Huang et al., indicating a common feature among GOC cases [11, 12, 16].

As summarized in Table 3, the radiographic profile of our five mandibular GOC cases aligns closely with the characteristic patterns already documented—multilocularity with straight septa, well-defined corticated borders, and frequent expansion or canal displacement—thus reinforcing both the diagnostic reliability of these imaging hallmarks and the need for vigilant, long-term radiographic surveillance after treatment [1, 2, 4–6].

## 4. Conclusion

This case series provides valuable insights into the radiographic and histopathological features of GOCs in the mandible. Male patients between the ages of 32 and 50 were documented in all five cases. Radiographic interpretation of the lesions appeared well defined, unilocular, and multilocular with corticated borders. Multilocular lesions display straight, long septa crossing at right angles, and scalloping between roots was noticeable. The aggressive nature of GOC was further demonstrated by the observation of root resorption, displacement of the mandibular canal and neighboring teeth, thinning and perforation of cortical borders, and extension across the midline.

Histopathologically, the lesions displayed nonkeratinized stratified squamous epithelium of variable thickness, hobnail cells, microcysts, mucoid/goblet cells, cilia, and epithelial spheres—consistent with some criteria proposed by Kaplan et al. The similarity in features among different odontogenic cysts and neoplasms underscores the significance of thorough histopathological examination. Inflammatory alterations and sclerotic bone reaction in one case further underline the risk for secondary infection. Considering the chance for recurrence and diagnostic challenges, correct diagnosis and long-term follow-up are crucial for the successful management of GOC.

## Figures and Tables

**Table 1:** The major and minor criteria listed by Kaplan et al. [11].

**Major criteria**	**Minor criteria**
1. Squamous epithelial lining with a flat interface with the connective tissue wall lacking basal palisading	1. Papillary proliferation of the lining epithelium
2. Epithelium exhibiting variations in thickness along cystic lining with or without epithelial spheres or whorls or focal luminal proliferation	2. Ciliated cells
3. Cuboidal eosinophilic cells or “hobnail” cells	3. Multicystic or multiluminal architecture
4. Mucous (goblet) pools, with or without crypts lined by mucous-producing cells	4. Clear or vacuolated cells in the basal or spinous layers
5. Intraepithelial glandular, microcystic, or duct-like structures	

**Table 2:** The histopathologic findings reported for the five cases in the current study.

**Histopathological criteria**	**Case # 1**	**Case # 2**	**Case # 3**	**Case # 4**	**Case # 5**
Major criteria					
Hobnail cells			✓	✓	
Variable epithelial thickening	✓	✓	✓	✓	✓
Microcysts		✓	✓		
Mucoid/goblet cells	✓		✓		
Apocrine snouting	✓			✓	
Epithelial spheres	✓	✓	✓		✓
Minor criteria					
Clear cells, cholesterol clefts	✓				
Papillary projections	✓	✓			
Cilia	✓	✓	✓		
Multiple cysts		✓	✓		

**Table 3:** Comparative radiographic and histopathological features of reported glandular odontogenic cyst cases.

**Study**	**Year**	**Cases**	**Sex**	**Site**	**Locularity/border**	**Specific radiographic features**	**Effects on structures**	**Histopathology**	**Recurrence**
Gardner et al.	1988	10	9M/1F	Mand > max	Unilocular 6; multilocular 4; corticated border	Scalloping between roots; well-defined margins	Expansion, displacement, root resorption 30%	Mucous, ciliated, hobnail cells; ducts	30%
Fowler et al.	2011	46	26M/20F	max 33%	Unilocular 52%; multilocular 48%	Scalloped, sometimes ill-defined; septa frequent	Expansion 78%; perforation 44%; resorption 36%	Mucous 100%, hobnail 93%, crypts 83%	21%
Maruyama et al.	2021	12	7M/5F	Mand 75%	Multilocular 58%	Fine septa; corticated margin	Expansion 100%; canal displacement 42%	Classic GOC epithelium	25%
Udompatanakorn et al.	2023	1	37M	Ant. mand	Unilocular	Smooth cortical outline	Expansion with cortical thinning	Typical epithelium; S100+, CK19+	—
Kochaji et al.	2023	1	48F	Post. mand	Multilocular	Straight septa intersecting at 90°	Canal displacement; resorption	Classic features	—
Current series (Sabban et al.)	2025	5	5M	Mand 100% (3 ant/2 post)	Unilocular 2; multilocular 3	Straight long septa at right angles; scalloping	Expansion; canal displacement; resorption 1; perforation 1	Mucous and hobnail cells; microcysts	n/a

## Data Availability

The data that support the findings of this study are available on request from the corresponding author. The data are not publicly available due to privacy or ethical restrictions.
